# Can Personalized 3D Kinematic Modeling Predict Loss of Pronation and Supination in Diaphyseal Forearm Malunions? A Clinical Validation Study

**DOI:** 10.1097/CORR.0000000000003945

**Published:** 2026-05-08

**Authors:** Derek F. R. van Loon, Eline M. van Es, Mark F. Siemensma, Denise Eygendaal, Filip Stockmans, DirkJan H. E. J. Veeger, Joost W. Colaris

**Affiliations:** 1 Department of Orthopaedics and Sports Medicine, Erasmus MC, University Medical Center Rotterdam, Rotterdam, the Netherlands; 2 Ortho Clinic Brugge, Kortrijk, Belgium; 3 Department of Biomechanical Engineering, Delft University of Technology, Delft, the Netherlands

## Abstract

**Background:**

Despite the clinical relevance of forearm fractures and malunions and the impact of a functional limitation, the link between forearm malalignment and limited pronation and supination remains poorly understood and still relies on anatomical alignment expressed as angulation. Using recently developed technologies, mechanisms that limit function can be automatically detected by modeling individual forearm kinematics using three-dimensional (3D) bone models of the radius and ulna.

**Questions/purposes:**

We evaluated the accuracy of a personalized 3D kinematic model to identify limitations in forearm rotation in pronation and supination and to answer the following questions: (1) How accurately does the model-predicted ROM agree with the corresponding clinical measurements? (2) How accurately does the model classify malunited forearms according to the presence of clinically relevant functional limitations, defined as a range of pronation or supination less than 50°? (3) What is the frequency at which the model detects bone impingement and central band block during pronation and supination?

**Methods:**

This retrospective study evaluated a diagnostic model using the preoperative CT scans of 45 patients with unilateral diaphyseal forearm malunions, all of whom underwent corrective osteotomy due to a clinically relevant limitation in pronation or supination function. In all, 53% (24) of patients were male; the mean ± SD age at the time of the CT scan was 16 ± 6 years, and the mean time since the original trauma was 6 ± 5 years. Twenty patients had a clinically relevant loss of pronation, 15 patients had a loss of supination, and 10 patients had a loss of both. We generated 3D bone models with landmarks to simulate forearm rotation in 5° steps from 100° of pronation to 100° of supination. Two mechanisms that limit function after diaphyseal malunions—bone impingement and central band blockage—were identified in the simulation, resulting in a predicted ROM. For the first study question, differences between clinical and predicted function were expressed as mean absolute error, root mean square error, and mean error to illustrate typical error size, penalize outliers, and quantify the direction of error deviation, respectively. Acceptable errors were around 15°, comparable to the range seen in clinical measurements. For question two, clinical measurements and predictions were dichotomized based on a threshold of 50°. Accuracy, sensitivity, specificity, positive and negative predictive values, and the area under the receiver operating characteristic (ROC) curve for detecting clinically relevant limitations were calculated separately for pronation and supination. Acceptable diagnostic values should be above 60%, which is normal for angulation measurements. For question three, the blocking mechanisms detected during the simulation were counted.

**Results:**

Mean absolute errors between prediction and clinical measurement for pronation, supination, and ROM were 19°, 23°, and 22°, respectively. Root mean square errors were 22° for pronation, 28° for supination, and 28° for ROM. Mean errors were 3° for pronation, 1° for supination, and 5° for ROM. Errors were substantially higher than the clinical measurement uncertainty, with some outliers. Accuracy for finding a relevant pronation or supination limitation was 91% and 82%, respectively. Diagnostic values for detecting pronation limitations were 91% for accuracy, 87% for sensitivity, 100% for specificity, 79% for negative predictive value, and 100% for positive predictive value. For supination, the values were 82% for accuracy, 84% for sensitivity, 80% for specificity, 80% for negative predictive value, and 84% for positive predictive value. Area under the curve values were 0.97 (95% confidence interval [CI] 0.93 to 1) for detecting pronation limitations and 0.93 (95% CI 0.87 to 1) for supination limitations. These values are higher than those reported by studies using angulation thresholds. Bone impingement was mainly seen during pronation, and a central band block was the most common reason for a supination limitation.

**Conclusion:**

Individualized kinematic modeling of forearm malunions reliably detects clinically relevant limitations of forearm rotation without requiring dynamic imaging. Because of simplifications on the exact location and status of the central band and the neutral position of the forearm, exact ROM prediction is not possible

**Clinical Relevance:**

This study represents an important step toward functional rather than anatomical evaluation of forearm anatomy and correction of malunited forearm fractures. The next step would be to use the model in preoperative planning optimization, focusing on functional outcomes rather than purely anatomical correction. Given the model’s high diagnostic accuracy, personalized 3D kinematic modeling has potential as a decision tool for determining whether a forearm fracture should undergo operative treatment or whether it can be managed nonoperatively. However, challenges regarding fracture remodeling and stability in a cast, along with low-dose 3D imaging, must be addressed.

## Introduction

Forearm fractures are the most common fractures among children, accounting for around 40% of all pediatric fractures [23, 30]. In contrast to adults, pediatric diaphyseal forearm fractures are frequently managed nonoperatively with cast immobilization because younger patients often are capable of substantial remodeling [10, 20]. Treatment decisions in the acute setting are therefore guided by patient factors such as age and sex, which influence remodeling potential, and by the degree of anatomical alignment, expressed as angulation, which influences the amount of remodeling needed [7, 25, 27]. Pronation and supination are essential movements for daily activities, such as eating, writing, and personal care, and unrestricted rotation is critical during childhood when motor skills and independence are developing [37]. Clinically, a range of pronation or supination less than 50° is considered a relevant restriction, as it can markedly impair function [19]. Yet the degree of angulation alone is a poor predictor of functional outcome: Patients with similar deformities may experience different levels of motion loss [6, 35]. Thus, although angulation is used for decision-making in acute fracture management, it offers little diagnostic value once healing has occurred. Despite the clinical relevance of forearm fractures and malunions and the impact of functional limitation, the link between forearm malalignment and forearm function remains poorly understood.

Recent research on the topic of three-dimensional (3D) imaging of malunited forearms has identified two causes of impaired forearm rotation related to changes in the distance between the radius and ulna [1, 14]. If the interosseous space becomes too narrow, the bones collide during rotation, resulting in bone impingement. This mechanism is most often seen during pronation because the distance between the bones diminishes to make the rotation of the radius around the ulna possible. Conversely, if the space becomes too wide, the tensioned central band of the interosseous membrane can act as a restraint, resulting in a central band block. This mechanism is most often seen during supination, as the distance between the bones increases, rotating the radius to lie parallel to the ulna. The most straightforward approach to differentiate between bone impingement and central band block would be dynamic imaging [1]. However, dynamic techniques are not always available, they expose patients to considerable radiation when using CT, they have low resolution when using MR, and they are labor-intensive to analyze [8, 11, 34, 42]. An alternative method, based on static 3D imaging, could model individual forearm kinematics with 3D bone models of the radius and ulna. Previous work has demonstrated automatic identification of important bony landmarks on 3D models of the radius and ulna [39]. Two landmarks can construct a validated rotation axis and can reliably model pronation and supination in unaffected forearms [38]. Combining these methods results in a fully automatic, personalized kinematic model of the forearm. However, the validity of modeling rotation in malunited forearms has not been tested. If forearm function can be reliably predicted directly from 3D models, clinicians would have an accurate and practical tool to assess the link between forearm shape and functional outcome.

We evaluated the accuracy of a personalized 3D kinematic model to identify limitations in forearm rotation in pronation and supination and to answer the following questions: (1) How accurately does the model-predicted ROM agree with the corresponding clinical measurements? (2) How accurately does the model classify malunited forearms according to the presence of clinically relevant functional limitations, defined as a range of pronation or supination less than 50°? (3) What is the frequency at which the model detects bone impingement and central band block during pronation and supination?

## Patients and Methods

### Overview of Study Design

This was a single-center, retrospective, validation study conducted at a large Dutch urban, tertiary referral academic medical center. We used baseline data from two prospective studies registered in the Dutch Trial Register (NL6324 and NL8059) on corrective osteotomies for malunited forearm fractures, one of which has already been published [32]. The datasets from the two studies were deemed suitable for our analyses because of the relatively liberal inclusion criteria, which were primarily focused on functional loss.

### Participants

Between October 2016 and October 2023, we treated 45 patients for diaphyseal forearm malunions. Of those, we considered as potentially eligible those who (1) had a malunion after a diaphyseal both-bone forearm fracture, (2) exhibited a clinically relevant loss of rotational function of the forearm, (3) reported symptoms related to their forearm, and (4) were at least 6 years old. The diaphysis was defined as the part of the bone between 20% and 80% of its total length [21]. A clinically relevant loss of rotational function was defined as having a pronation or supination range less than 50° [19]. We excluded patients with (1) a nonanatomical shape of the contralateral forearm based on congenital or acquired abnormalities and (2) a nonanatomical shape of the affected arm based on congenital or acquired nontraumatic abnormalities. Patients were included in the two studies mentioned previously (see “Overview of Study Design”). Therefore, all 45 eligible patients were included. Because only baseline data were used, no patients were lost to follow-up or had incomplete datasets.

### Descriptive Data

All 45 included patients were eligible for analysis. The mean ± SD age at the time of scanning was 16 ± 6 years. The mean ± SD time between trauma and scan was 6 ± 5 years. Fifty-three percent (24) of the participants were male. We did not perform a by-sex analysis in this study because there is no rationale for different expected outcomes in functional forearm malunions. A relevant loss in pronation and supination (range < 50°) was measured in 22% (10) of patients, whereas 44% (20) of patients had a relevant loss of pronation and 33% (15) had a relevant loss of supination (Table 1).

**Table 1.** Patient characteristics

Parameter	Total (n = 45)
Sex, male	53 (24)
Age at CT scan in years	16 ± 6
Time between trauma and scan in years	6 ± 5
Left arm involved	53 (24)
Dominant arm involved	51 (23)
Patients with only a clinically relevant pronation loss^a^	44 (20)
Patients with only a clinically relevant supination loss^a^	33 (15)
Patients with clinically relevant pronation and supination loss^a^	22 (10)
Pronation, unaffected side in °	76 ± 8
Supination, unaffected side in °	88 ± 5
ROM, unaffected side in °	164 ± 10
Pronation, affected side in °	41 ± 24
Supination, affected side in °	41 ± 33
ROM, affected side in °	82 ± 22

Data presented as % (n) or mean ± SD.

a Loss is defined as clinically relevant if the range of pronation or supination, or both, is less than 50°.

### Data Acquisition

**Fig. 1:**
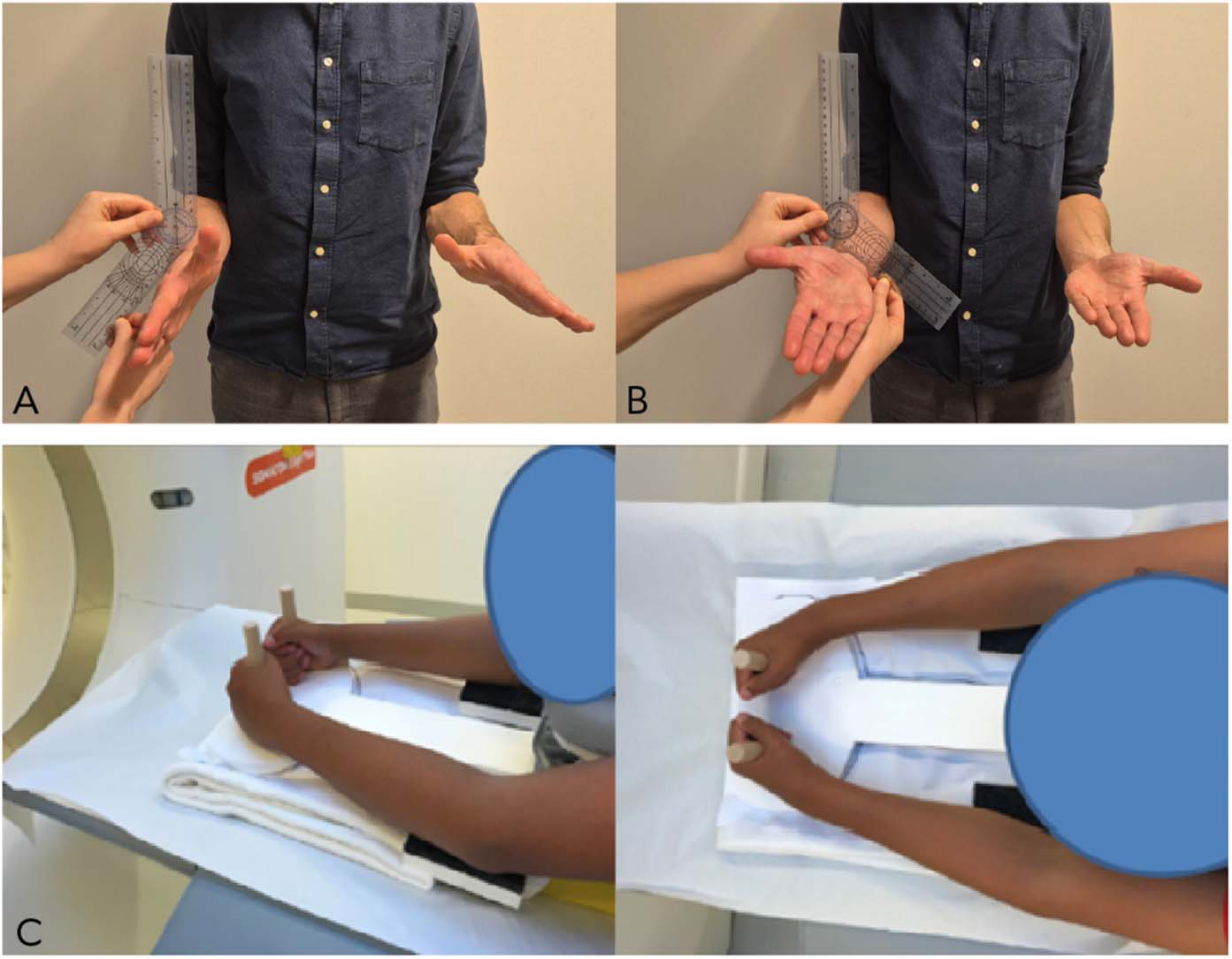
Examples of clinical measurements and scan protocol showing (**A**) the measurement of pronation and (**B**) the measurement of supination. (**C**) Shown here is a patient scanned in a CT scanner in the prone position with the custom holder.

Data acquisition included ROM measurements, a CT scan, and patient demographics. An experienced researcher (EMVE) measured the rotational function of both forearms with a universal goniometer following the protocol defined by the American Society of Hand Therapists [13]. The patient stood in an upright position with the elbow positioned against the torso to ensure no compensating movement from the shoulder or elbow. With the elbow in 90° of flexion, the forearm was placed in neutral rotation, with the thumb pointing upward. One arm of the goniometer was aligned with the upper arm. The other arm was placed parallel to the distal third of the volar side of the forearm to measure supination and subsequently placed to the dorsal side to measure pronation (Fig. 1A-B) [5]. Forearm function was expressed as the amount of pronation and supination, rounded to the nearest multiple of 5°. The function was negative if a forearm could not reach the neutral position. A CT scan of both forearms was performed with the patient in a prone position and their arms stretched above their head. Patients were asked to place their hands with palms facing each other during the scan to mimic a neutral position. When available, a holder with two upright sticks was used, which could be held during scanning to minimize movement (Fig. 1C). The patient demographics collected were age, age at trauma, sex, side of malunion, and dominant side.

### Kinematic Modeling

The radius and ulna were segmented from the CT scan using Materialise Mimics (Materialise NV), and 3D surface models were obtained. Before being exported as STL files, the 3D models were postprocessed using Materialise 3-Matic (Materialise NV). All steps of the kinematic modeling were performed using custom Python code, illustrated in a step-by-step animation (Supplemental Video Content; http://links.lww.com/CORR/B523). The only inputs required to start the kinematic modeling were the 3D models of the radius and ulna and whether the forearm was left or right.

**Fig. 2:**
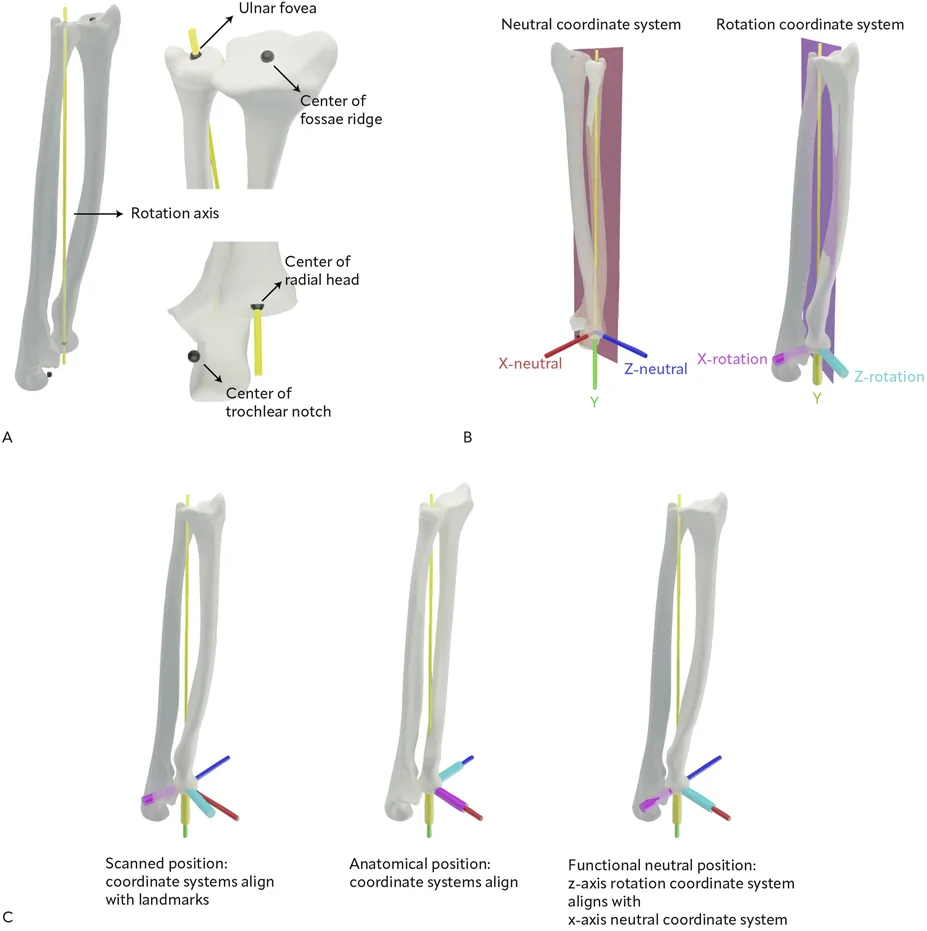
This figure demonstrates radial and ulnar landmarks, coordinate systems, and calculation of forearm positions. (**A**) Four landmarks were identified on each forearm, two at the wrist and two at the elbow. (**B**) Two coordinate systems were calculated: a neutral coordinate system based on the rotation axis and the trochlear notch and a rotation coordinate system based on the rotation axis and the fossae ridge. (**C**) Using the coordinate systems, the difference in scanned position can be seen. When the coordinate systems align, the forearm is in the anatomical position. When the z-axis of the rotation coordinate system aligns with the x-axis of the neutral coordinate system, the difference is 90° from the anatomical position, which is defined as the functional neutral position.

First, four landmarks were automatically located on the 3D models (Fig. 2A): On the radius, they are the center of the radial head and the center of the fossa ridge, and on the ulna, they are the center of the trochlear notch and the fovea [39]. Any improper landmark placements identified on visual inspection were corrected manually. Second, the landmarks at the center of the radial head and the ulnar fovea were used to reconstruct a rotation axis (Fig. 2A) [38]. This rotation axis has been shown to accurately describe forearm rotation in unaffected forearms. Third, the scanned position of the forearm was calculated. Because the scan protocol allowed for forearm rotation from the shoulder and elbow, the scanned position of the forearm was not equal to the neutral position. Therefore, a scanned forearm position was calculated to define the neutral position. The functional neutral position was defined as a 90° difference from the anatomical position, which is the forearm in maximal supination. To calculate the difference of the scanned position relative to the functional neutral position, two coordinate systems around the rotation axis were defined: one neutral coordinate system based on the rotation axis and the proximal ulna and the rotation coordinate system based on the rotation axis and the distal radius (Fig. 2B). When the rotation coordinate system aligns with the neutral coordinate system, the forearm is in the anatomical position. By calculating the transformation that aligns the rotation coordinate system with the neutral coordinate system, the angle between the two can be retrieved. By defining that neutral forearm rotation differs 90° from the anatomical position, the scanned position is found by subtracting 90° from the found transformation (Fig. 2C). These steps were computed using established equations (Appendix 1; http://links.lww.com/CORR/B522). The scanned position of all individuals was recorded (Appendix 2; http://links.lww.com/CORR/B522). Forearm rotation was modeled by rotating the radius around the rotation axis in 5° increments from 100° of supination to 100° of pronation. We chose 100° to match the maximal measured function of the unaffected forearms. Thus, rotation was modeled in 41 positions of 5°, of which one was equal to the forearm position in the CT scan. Because rotation was modeled in steps of 5°, the found scanned position was also first rounded to the nearest multiple of five. It was assumed that rotation equaled function: If a 5° supination rotation was applied to the scanned position, the acquired modeled position was equal to the scanned position plus 5° of supination.

**Fig. 3:**
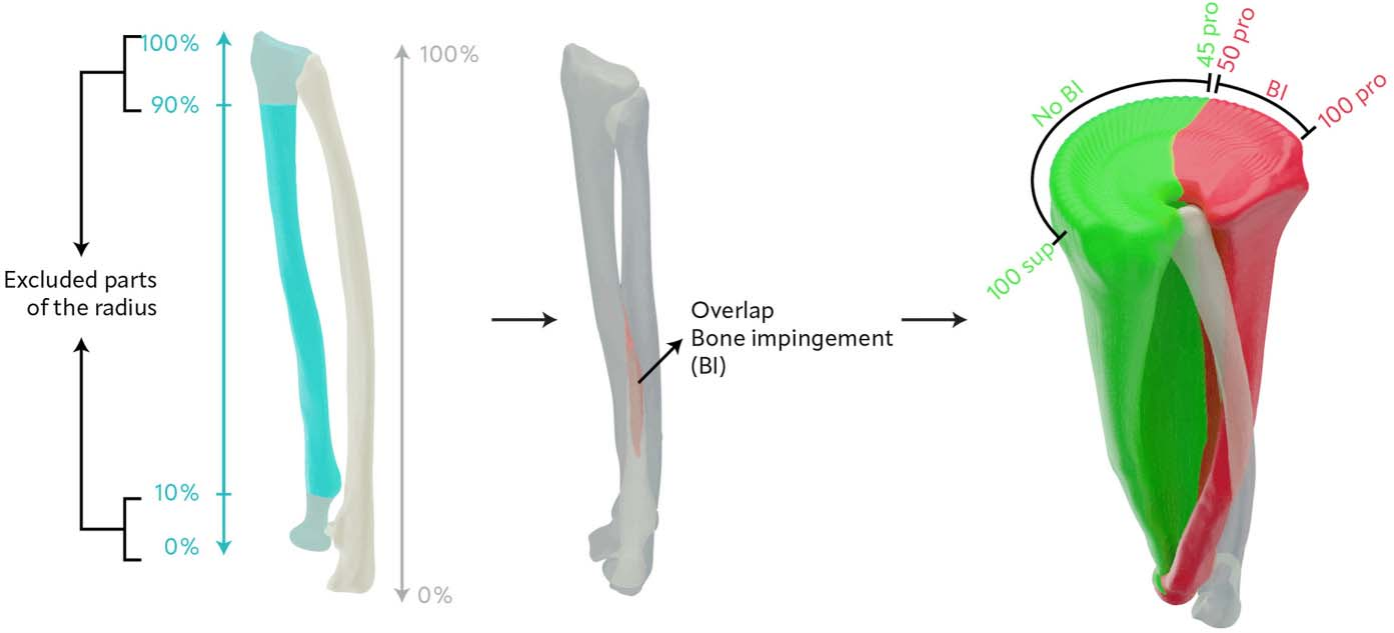
This image shows how bone impingement is detected for each forearm position. Ten percent of the proximal and distal parts of the radius were excluded from the analysis. If the diaphysis of the radius overlaps the ulna in a position, this indicates bone impingement. BI = bone impingement; sup = supination; pro = pronation.

**Fig. 4:**
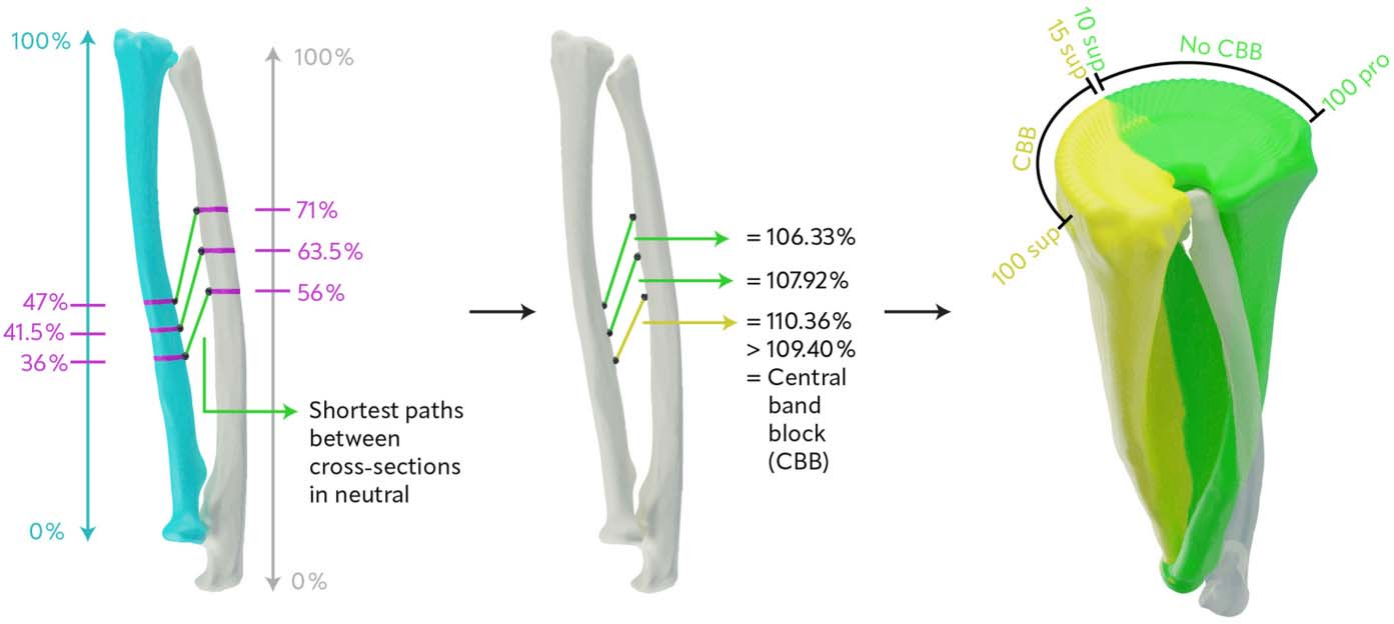
This image shows how the central band and its length are measured. In the neutral position, the shortest path between three cross sections of the radius and ulna is calculated, indicating the proximal, middle, and distal parts of the central band. If the relative length of the proximal, middle, or distal central band is longer than 109.4%, 108.4%, or 107.9% respectively, compared to its shortest length during rotation, this indicates a central band block. CBB = central band block; sup = supination; pro = pronation.

To check for bone impingement, we calculated whether the radius overlapped the ulna in each of the 41 forearm positions. This calculation excluded the elbows and wrists, defined as the most proximal and distal 10% of the radius model (Fig. 3). To check for central band block, we calculated the lengths of the central band in each of the 41 forearm positions and compared them to the shortest lengths during rotation. To measure central band length, the origin and insertion of the central band on the radius and ulna were identified based on previous research [24]. Using the 3D bone models of the forearm in the neutral position, the shortest paths between the radius and ulna at three axial cross sections indicated the proximal, middle, and distal parts of the central band. The heights of the cross sections from proximal to distal retrieved from a previous study were 36.0%, 41.5%, and 47.0% of the radial length and 56.0%, 63.5%, and 71.0% of the ulnar length (Fig. 4) [24]. The shortest path between two cross sections yields three sets (proximal, middle, and distal) of two points, one on the radius and one on the ulna, which are fixed at these locations on the bone models. At different forearm positions, the distance between these points indicates the length of a part of the central band. The relative length of the central band is calculated by dividing the length of the central band in each forearm position by the shortest length measured across all positions, excluding those where bone impingement is detected. The threshold for excessive central band length is based on modeling of the unaffected contralateral forearms from our dataset. The maximum relative length for the proximal, middle, and distal segments was determined. The thresholds were 109.4%, 108.4%, and 107.9% for the proximal, middle, and distal segments, respectively. Thresholds were rounded at 0.1% because of the small changes observed between rotational positions in the modeling of unaffected forearms, consistent with other research [12, 18]. Data and calculations of central band length thresholds were recorded (Appendix 3; http://links.lww.com/CORR/B522).

### Primary and Secondary Study Outcomes

Our first study goal was to calculate the model’s accuracy in predicting the range of pronation, supination, and ROM. To achieve this, the differences between predicted and measured ROM, pronation, and supination were calculated. These differences were expressed as three error scores: the mean absolute error, root mean square error, and mean error. Because the model can produce under- or overestimations, the mean absolute error between measurement and prediction only considers the magnitude of the prediction error and thus reflects the typical size of the model’s prediction error. By taking the root of the squared errors, the direction of the error is neglected, and larger errors are penalized more. If this root mean square error is larger than the mean absolute error, this shows that the model can produce outliers and exhibit variability in error size. If the errors are averaged on a face-value basis, the direction and magnitude of the typical over- or underestimation are given by this mean error. Acceptable absolute and root mean square errors would be 15°, matching the published variability in clinical measurements of pronation and supination [3, 5, 29]. Our second study goal was to calculate the model’s accuracy in predicting clinically relevant limitations. Model predictions were dichotomized to indicate a clinically relevant limitation when the predicted range of pronation or supination was less than 50°, and the results were compared with the dichotomized clinical measurements. Accuracy values of our model must improve over standard diagnostic methods for measuring angulation to determine whether a diaphyseal forearm fracture or malunion will lead to a limitation. These reference values vary with what is used in clinical practice, but accuracies of 60% for measuring fracture angulation on radiographs and 80% for 3D analysis of malunions are used as our thresholds [4, 35]. Our third study goal was to determine how often a limitation mechanism occurs during either pronation or supination. Bone impingement and central band block were considered when they were seen during pronation or supination. Results were categorized into blocking mechanisms that led to a clinically relevant limitation, thus seen below the 50° threshold, and those that did not, thus seen between 50° and 100°.

### Ethical Approval

The Medical Ethical Committee of the Erasmus MC provided ethical approval of the studies (MEC-2015-606 and MEC-2019-0025). Protocols of both studies were registered in the Dutch Trial Register (NL6324 and NL8059). All patients provided informed consent and agreed for their data to be used for further research.

### Statistical Analysis

**Fig. 5:**
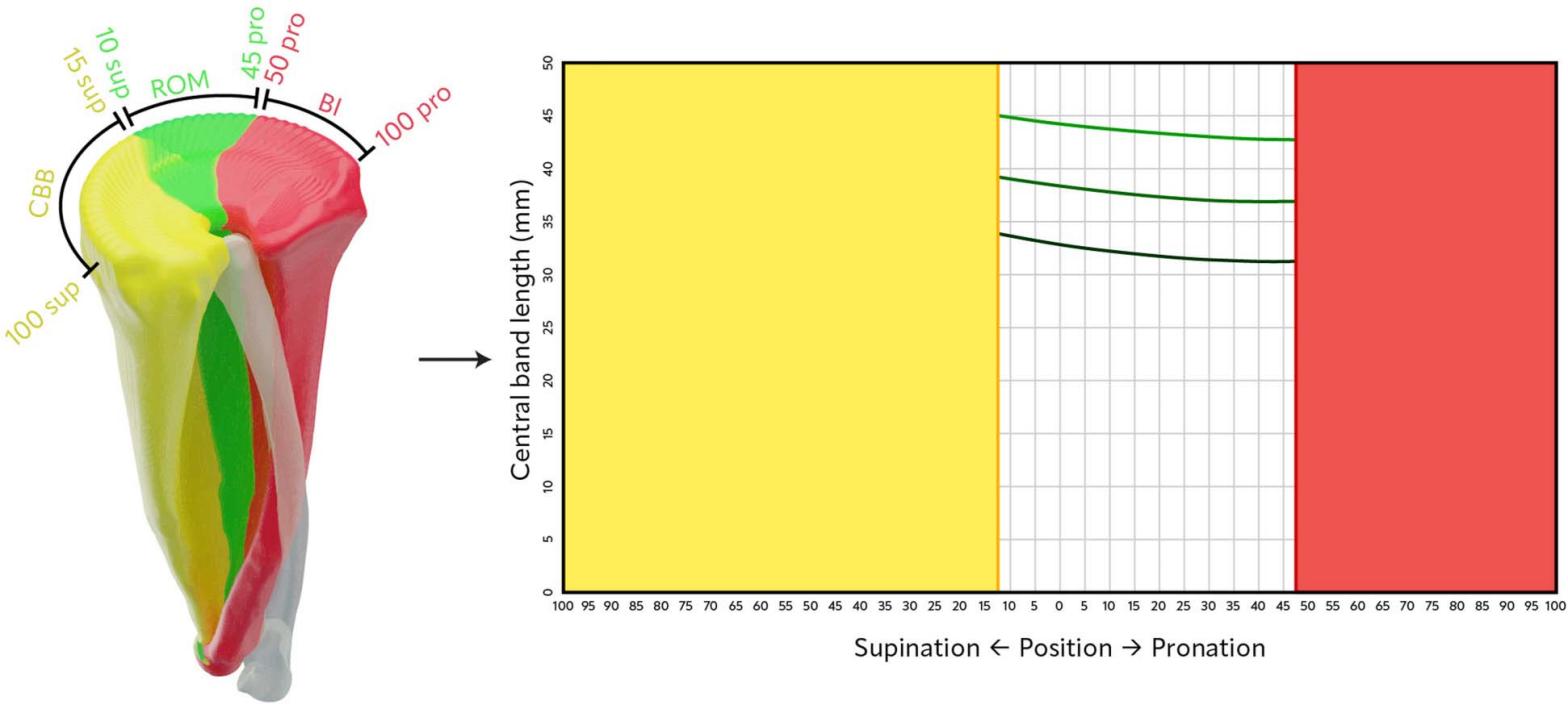
This image shows how the combined measurements of bone impingement and central band block lead to a prediction of the ROM. To the left, the combined blocking mechanisms are shown for one patient. These data are then presented in a graph on the right, with the positions of the forearm on the x-axis and the length of the central band on the y-axis. The green lines indicate the length of the central band’s proximal, middle, and distal parts. The yellow area indicates a central band block, and the red area indicates bone impingement. CBB = central band block; BI = bone impingement; sup = supination; pro = pronation.

All outcome measures and model performance scores were calculated in R version 4.5.1 [28]. Each forearm’s maximal ROM, maximal pronation, and maximal supination were predicted by combining bone impingement and central band block (Fig. 5). Errors were calculated by subtracting the predicted function from the measured function. Accuracy, sensitivity, specificity, positive predictive value (PPV), negative predictive value (NPV), and area under the curve (AUC) of the receiver operating characteristic (ROC) curve were calculated using the pROC package [31]. The 95% confidence intervals (CIs) for accuracy, sensitivity, specificity, PPV, and NPV were estimated via bootstrapping, whereas the AUC was estimated using the DeLong method. The PPV and NPV were based on the prevalence of a limitation in the affected forearms.

## Results

### Agreement Between Model-predicted ROM and Corresponding Clinical Measurements

**Fig. 6:**
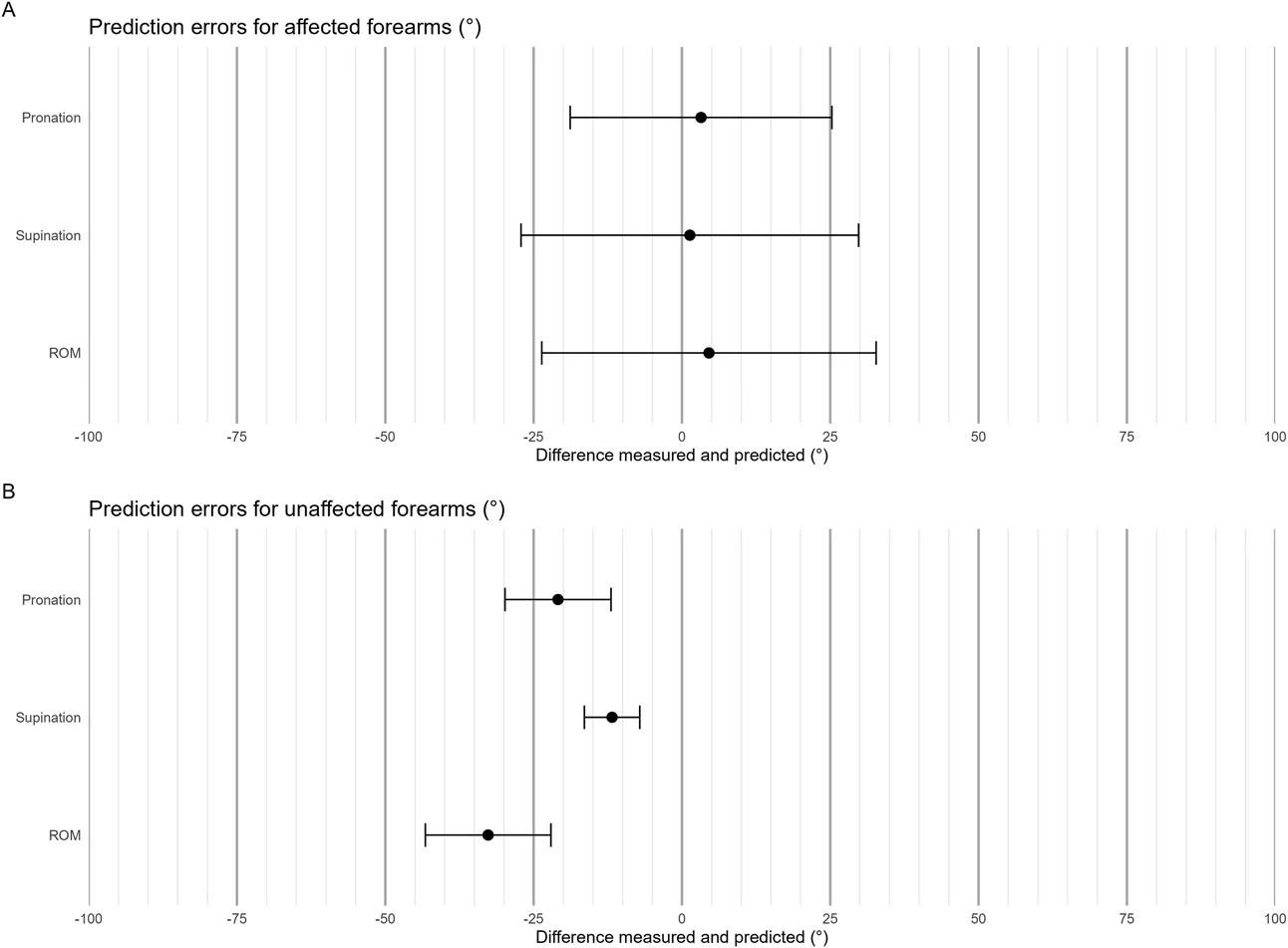
Plots showing the mean prediction errors (measured-predicted) and standard deviation for pronation, supination, and ROM for (**A**) affected forearms and (**B**) unaffected forearms.

For the affected forearms, mean absolute errors were 19° for pronation, 23° for supination, and 22° for ROM, indicating large errors for predicting function (Table 2). The root mean square errors were higher than the mean absolute errors, indicating the presence of outliers and large variance between the errors. The mean errors were 3° for pronation, 1° for supination, and 5° for ROM, indicating that the model slightly underestimates function (Fig. 6A).

**Table 2.** Error of the predicted function

Error	Pronation	Supination	ROM
Affected forearms			
Mean absolute error ± SD	19° ± 12°	23° ± 17°	22° ± 18°
Root mean square error	22°	28°	28°
Mean error ± SD	3° ± 22°	1° ± 28°	5° ± 28°
Unaffected forearms			
Mean absolute error ± SD	21° ± 9°	12° ± 5°	33° ± 11°
Root mean square error	23°	13°	34°
Mean error ± SD	-21° ± 9°	-12° ± 5°	-33° ± 11°

Mean absolute error reflects the typical prediction error. The root mean square error shows whether outliers are present if the value is larger than mean absolute error. The mean error shows systematic underestimation (if positive) or overestimation (if negative) of the model.

For the unaffected forearms, the mean absolute error was 21° for pronation, 12° for supination, and 33° for ROM (Table 2). The root mean square errors were 23° for pronation, 13° for supination, and 34° for ROM, and they were almost equal to the mean absolute errors, indicating small variability in error size. The mean errors were equal in magnitude to the mean absolute errors but were negative, indicating that in every case, the model overestimated forearm function (Fig. 6B). This was as expected because the limits on central band length were set based on these measurements, and we expected no bone impingement in these unaffected forearms.

All individual clinical measurements and predictions of functions were recorded (Appendix 4; http://links.lww.com/CORR/B522).

### Model Accuracy for Classifying Malunited Forearms

**Fig. 7:**
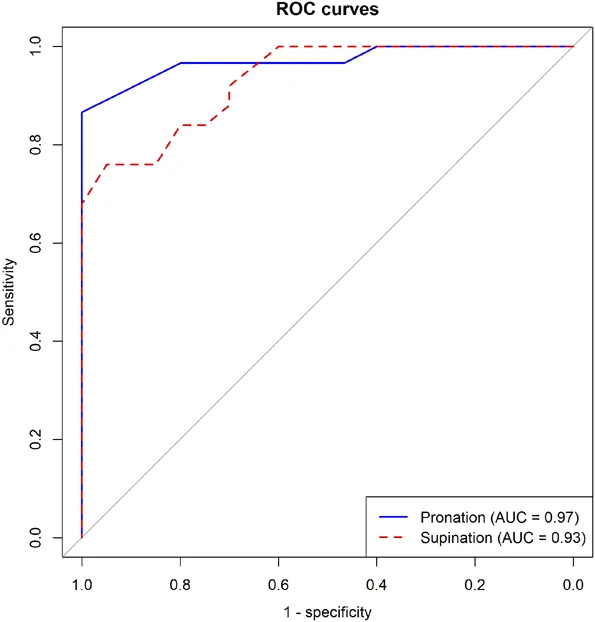
ROC curves for recognizing a relevant pronation and supination limitation.

The accuracy for finding a relevant pronation or supination limitation was 91% and 82%, respectively (Table 3). Values were calculated based on the confusion matrices (Table 4). The diagnostic performance measures for pronation limitations were therefore almost all higher than supination limitations: 87% for sensitivity, 100% for specificity, 100% for PPV, and 79% for NPV. All diagnostic values for recognizing a relevant supination limitation were lower, except for the NPV; the values were 84% for sensitivity, 80% for specificity, 84% for PPV, and 80% for NPV. The AUC indicated very good reliability, at 0.97 (95% CI 0.93 to 1) for pronation limitations and 0.93 (95% CI 0.87 to 1) for supination limitations (Fig. 7) (remember that when interpreting an AUC, perfect overall ability to distinguish between patients with and without limitations would be represented by 1.0, and complete randomness would have an AUC of 0.5).

**Table 3.** Diagnostic values for recognizing clinically relevant limitations (< 50°) and the 95% confidence intervals

Diagnostic value	Pronation limitation	Supination limitation
Accuracy	91 (82-98)	82 (69-93)
Sensitivity	87 (73-97)	84 (68-96)
Specificity	100 (100-100)	80 (60-95)
Positive predictive value	100 (100-100)	84 (72-96)
Negative predictive value	79 (65-94)	80 (65-94)
Area under the curve	0.97 (0.93-1)	0.93 (0.87-1)

Data presented as % (95% CI).

**Table 4.** Confusion matrices for pronation and supination limitations

Clinically relevant pronation limitations		Measured	
		No limitation	Limitation
Predicted	No limitation	15	4
	Limitation	0	26

Clinically relevant supination limitations		Measured	
		No limitation	Limitation
Predicted	No limitation	16	4
	Limitation	4	21

### Frequency of Detecting Bone Impingement and Central Band Block During Pronation and Supination

In the malunited forearms, bone impingement was most often observed during pronation (Fig. 5), occurring 35 times compared to 5 instances of a central band block (Table 5). The opposite was true for supination: bone impingement occurred 2 times, whereas a central band block occurred 33 times. None of the bone impingement occurrences led to a relevant supination loss; however, in one forearm, a central band block was predicted to result in a pronation loss. In the unaffected forearms, bone impingement was seen 11 times, but none of the occurrences led to a relevant limitation. Central band blocks were never seen because the thresholds for a central band block are based upon these forearms. All blocking mechanisms leading to the individual predictions were recorded (Appendix 4; http://links.lww.com/CORR/B522).

**Table 5.** Number of times a blocking mechanism is seen for each limitation in malunited forearms

Function and range	Bone impingement (n = 37)	Central band block (n = 38)
Pronation	95 (35)	13 (5)
> 50°-100°	27 (10)	11 (4)
< 50°	68 (25)	3 (1)
Supination	5 (2)	87 (33)
> 50°-100°	5 (2)	24 (9)
< 50°	0 (0)	63 (24)

Data presented as % (n). Values reflect limitations detected in a certain range of pronation and supination.

## Discussion

Improving the treatment of forearm fractures requires a clear understanding of how bone shape influences rotational function. Research has identified two primary mechanisms restricting rotation in malunited forearms: bone impingement and a central band block, first identified in cadaveric studies and more recently in vivo [1, 15]. In this study, we aimed to take the next step by validating a personalized 3D model that predicts rotational forearm function in malunited fractures based on the patient’s bony anatomy and the presence of these two blocking mechanisms. The evaluation of our model revealed that clinically relevant rotational limitations can be accurately identified, with high diagnostic accuracy, in malunited forearms. Our model also found that bone impingement was the most common reason for a pronation limitation, whereas a central band block was the most common for a supination limitation. Although precise ROM predictions will require further refinement, including detailed knowledge of forearm kinematics and individualized adjustments, the current model is sufficient to differentiate between functional and nonfunctional forearm shapes based on anatomy alone. Therefore, we believe that the model has the potential to improve posttraumatic evaluation from anatomical alignment to functional outcomes. Kinematic modeling can guide corrective interventions and determine whether correcting only the radius or the ulna will yield satisfactory results. Only a central band release may be sufficient in some patients, as shown by the modeling. Furthermore, given its high diagnostic accuracy, it has potential as a decision tool for determining whether a forearm fracture requires reduction. However, there are still many challenges to consider, in addition to the current limitations in modeling. First, fracture instability, which influences shape changes until bone union, and correction due to growth, which influences shape changes after bone union in children, must be understood. If shape can be predicted, this model can then help to predict function. Second, standard diagnostics rely on two-dimensional radiographic images. Methods for retrieving 3D models with a low radiation dose should be developed to enable modeling of forearm rotation in larger populations.

### Limitations

This study has several limitations. First, the data only include unaffected forearms and malunions with clear functional restrictions. Malunions without severely impaired forearm rotation are not present in our dataset. Because these patients do not need treatment, 3D data on mild malunions without notable functional impairment are unavailable. Second, regarding the modeling, we relied on a rotation axis validated only for forearms without malunions. This assumption of normal kinematics in malunited forearms may be invalid in some instances. One study has shown that the kinematics of forearm rotation do not meaningfully change after a malunion of the distal radius, but there is no report on diaphyseal malunions [17]. To ensure that changes in kinematics do not influence the results, we simplified the modeling by excluding the most proximal and distal 10% of the radius when checking bone impingement. The consequence, however, is that the current model does not support recognizing changes or limitations in the proximal or distal radioulnar joint. More research on the topic of kinematic forearm modeling is needed to account for this as well. Third, the mechanism of function loss has not been validated with dynamic CT or MR in our population; however, we do not believe this is necessary for our diagnostic model. What matters is that either the space between the radius and ulna becomes too small or too large; bone impingement and central band block are measurable effects of these principles. Although the measurement or threshold for these mechanisms could be improved through dynamic imaging, it does not alter the use of the measurement as a diagnostic tool to distinguish between forearms with and without a clinically relevant limitation. Fourth, the same is true about the origin, insertion, status, and maximum length of the central band. This information was unknown in these patients and thus was calculated in a reference position for all patients. Although MRI or ultrasound can provide additional information, these techniques are not commonly used in daily clinics, and having this extra information available for diagnostics was deemed outside the scope of our model [9]. Of course, obtaining this information could improve the model and decision-making. Furthermore, although we could set the threshold for a central band block on a patient-specific basis by using the unaffected side for each patient, we chose to standardize the threshold across all patients. Finally, we did not adjust the threshold by sex or age due to the relatively small dataset. More knowledge about the development of the central band during growth and sex differences could improve modeling results.

### Agreement Between Model-predicted ROM and Corresponding Clinical Measurements

Our results show that a precise function prediction from the kinematic model is inaccurate. Large mean absolute errors ranging between 18° and 22° and root mean square errors between 22° and 28° for the affected forearms indicate large errors in predictions, meaning that these values cannot be used in practice. Although the mean errors are minor, ranging from 1° to 5°, these only suggest that there is no bias toward overestimation or underestimation. The large SDs of the mean absolute errors reveal the same uncertainty in predicting the function. The reason for these large errors is twofold: First, our kinematic model is a simplified representation. One simplification we made was assuming that the ratio between the rotation of the radius and the function was equal. However, this is not actually the case. Although most of the forearm rotation is due to the rotation of the radius, some translation and movements of the ulna also contribute to differences between function and radius rotation [22, 36, 41, 43]. Furthermore, the range of supination and pronation is based on the neutral position, which is defined only as a functional reference pose during physical examination and requires a fixed shoulder and elbow position. By definition, when scanning the patient in the prone position, the shoulder and elbow are not in the defined positions. For this reason, and because the scanned position varied across patients, we generalized the model by adding a landmark-based method to determine the neutral position. This also had the advantage of allowing modeling of patients who are unable to achieve the neutral position. Second, the functional measurements done in the clinic are known to have considerable variability with large minimal detectable differences. A study with 20 raters on the accuracy of pronation and supination measurements on volunteers with unaffected arms reported a minimal detectable difference of 19°, a standard error of 7°, and an intraclass correlation coefficient of 0.35 for measuring full ROM, indicating poor agreement [29]. Two studies that considered only two raters reported lower minimal detectable differences: 7° for pronation, 6° for supination, and 10° for full ROM when measuring function in children [3, 5]. The intraclass correlation coefficients were also higher, ranging from 0.92 to 0.97 [3, 5]. Based on these studies, we would have accepted a mean absolute error and a root mean square error of 15° for pronation and supination. Our errors, however, are much larger, meaning that the ROM prediction is not particularly helpful in practice. We do believe that research focused on closing the gap between high variability in functional measurements and individualized forearm kinematics would be highly beneficial.

### Model Accuracy for Classifying Malunited Forearms

We found high diagnostic accuracy for detecting limitations in pronation (79% to 100%) and in supination (80% to 84%). These values, in combination with high AUCs of 0.97 and 0.93 for pronation and supination, respectively, reveal an excellent ability to detect functional limitations, despite large errors in ROM prediction. No clinically relevant functional limitations are predicted in the contralateral, unaffected forearms because they were used to set the thresholds for central band block, and bone impingement is not seen below 50° of pronation and supination. These results clearly distinguish modeling results between unaffected forearms and malunited forearms with and without clinically relevant limitations.

Although the binary groups of having or not having a clinically relevant loss are determined simply by a threshold of 50°, the accuracy of the diagnostic values is much higher than expected, considering the prediction errors. This is because of the clear differences in the pattern seen between unaffected forearms, affected forearms without clinically relevant limitations, and affected forearms with a clinically relevant limitation. Unaffected forearms show no central band block because of the thresholds in the current model, and bone impingement is only observed at extreme pronation in some patients. In the affected forearms, blocking mechanisms are seen in almost every forearm, but a clinically relevant limitation is not always predicted. The 50° threshold, used in practice to define relevant functional limitations, was therefore a logical choice in this model and led to good outcomes.

The standard diagnostic test to detect anatomical malalignment and guide treatment after fracture is angulation measurement on conventional radiographs. The predictive value of these measurements on functional limitations is low. A meta-analysis showed that a radius angulation of ≥ 15° on a lateral radiograph after trauma is an independent risk factor for developing a symptomatic malunion, at a rate of 46% [33]. The study with the largest population in this meta-analysis examined the association between angular malalignment and limitation of forearm rotation on 2D radiographs 7 months after fracture [4]. They found that a threshold of ≥ 16° angulation of the radius and/or ulna led to a 60% chance of developing a symptomatic malunion. A study focusing on the association between 3D angular malalignment and limitation found that for the radius, a dorsal angulation of 18° led to a sensitivity of 71% and a specificity of 74% for predicting a relevant pronation limitation [35]. These values are lower than our sensitivity of 87% and specificity of 100%. Their finding concerning a supination limitation is only linked to the axial deformity of the radius and ulna. The sensitivity is 71% and the specificity is 80%. The sensitivity is lower than our 84%, but our specificity of 80% is comparable. We believe our model outperforms the angulation measurements because it considers both the radius and ulna simultaneously. All studies that measure angulation attempt to correlate the angulation in one plane of a single bone to the loss of function, whereas our model considers the shapes of both bones. A multivariate regression model using the angulation of both bones across all three planes could be of value. Still, it would require large amounts of data to achieve sufficient statistical power.

### Frequency of Detecting Bone Impingement and Central Band Block During Pronation and Supination

In most cases, a limitation of pronation was due to bone impingement and a restriction in supination was due to a central band block. This finding is as expected because the space between the radius and ulna along the diaphysis becomes smaller during pronation than during supination. Conversely, the space widens during supination, leading to a blockage of the central band. This was already demonstrated more than 50 years ago, when the space between the bones during pronation and supination was measured [2]. In more recent research, this effect is described to have a limiting impact on rotation after a malunion. Still, it is always connected to the direction of the measured angulation of either the radius or ulna. It has been shown in patient studies that the length of the central band is larger in supination than in pronation in patients with a supination limitation, but only in forearms with a valgus deformity of the ulna [1]. Also in that study, bone impingement was seen in 15 of 16 patients in pronation when an extension deformity of the radius was present. Both the ulnar valgus deformity and radial extension deformity are the most common deformities seen among forearm malunions. Miyake et al. [16] explained that a fall on a stretched hand often leads to a clear angulation pattern, consisting of an extension deformity of the radius and a valgus deformity of the ulna. This pattern was observed in 76% (16) of patients in the same study [16] and in 89% (16) of another [1]. Although we have not measured angulation, we observed a similar pattern: predominantly bone impingement in pronation and a central band block in supination. These results explain the higher accuracy for detecting a pronation limitation compared to supination. The finding of bone impingement by our model is more certain than the central band block because it is solely dependent on the shape of the bone, which was accurately retrieved from the CT scan. The central band is based solely on averaged data from the evidence, and the thresholds are not age or sex controlled, resulting in decreased accuracy [24]. Alternatively, additional imaging, such as echography or MRI, or advanced techniques, such as statistical shape modeling, could provide a better solution [26, 40].

### Conclusion

Individualized kinematic modeling of forearm malunions reliably detects clinically relevant limitations without requiring dynamic imaging. Because of simplifications on the exact location and status of the central band and the neutral position of the forearm, an exact ROM prediction is not possible. More research is needed to understand forearm kinematics and central band changes after forearm malunions to improve ROM predictions.
